# Design and Synthesis of 4-O-Podophyllotoxin Sulfamate Derivatives as Potential Cytotoxic Agents

**DOI:** 10.1155/2021/6672807

**Published:** 2021-01-25

**Authors:** Ammar Bader, Majdi M. Bkhaitan, Ashraf N. Abdalla, Qasem M. A. Abdallah, Hamed I. Ali, Dima A. Sabbah, Ghadeer Albadawi, Ghassan M. Abushaikha

**Affiliations:** ^1^Umm Al-Qura University, Makkah 21955, Saudi Arabia; ^2^Biomedical Sciences Unit, Arab American University, Jenin, State of Palestine; ^3^Department of Pharmacology and Toxicology, College of Pharmacy, Taif University, Taif, Saudi Arabia; ^4^Faculty of Pharmacy and Medical Sciences, University of Petra, Amman, Jordan; ^5^Rangel College of Pharmacy, Health Science Center, Texas A&M University, Kingsville, TX 78363, USA; ^6^Department of Pharmacy, Al-Zaytoonah University of Jordan, P.O. Box 130, Amman 11733, Jordan

## Abstract

4-O-Podophyllotoxin sulfamate derivatives were prepared using the natural lignan podophyllotoxin. The prepared compounds were afforded by reacting O-sulfonyl chloride podophyllotoxin with ammonia or aminoaryl/heteroaryl motif. Biological evaluation was performed in human breast cancer (MCF7), ovarian cancer (A2780), colon adenocarcinoma (HT29), and normal lung fibroblast (MRC5) cell lines. Compound **3** exhibited potent inhibitory activity and good selectivity margin. Compounds **2**, **3,** and **7** exerted apoptotic effect in MCF7 cells in a dose-dependent manner. The cytotoxicity of the verified compounds was inferior to that of podophyllotoxin.

## 1. Introduction

Podophyllotoxin 1 (PPT) is a natural lignan of the aryl tetralin class and the main component of *Podophyllum peltatum* L., and it was used in folk medicine since remote times [1, 2]. It is generally considered as an effective anticancer agent with the ability to inhibit microtubules assembly by interacting with the tubulin proteins at the same binding site of colchicine, preventing the formation of the mitotic spindle [3, 4].
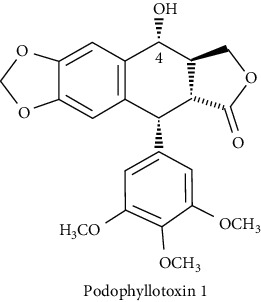


Serious adverse effects, including nausea, vomiting, diarrhea, abdominal pain, thrombocytopenia, leucopenia, cytotoxicity, and lack of oocytes maturation, were documented for PPT [5–7]. In addition, PPT suffers from poor water solubility and poor pharmacokinetic properties, limiting its wide spread in clinical use [8]. Nevertheless, due to its extraordinary biological activity and longtime use in traditional medicine, PPT and 4′-demethylpodophyllotoxin will continue to represent an important lead in the development of new active entities [9–13].

**Figure 1:**
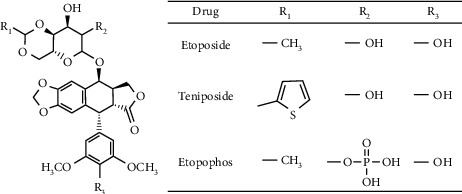
Podophyllotoxin derivatives in clinical use.

Multiple structural modifications were made on podophyllotoxin structure to improve the potency and pharmacokinetic properties as well as to overcome drug resistance. In particular, the C-4 position has represented a primary target for these modifications [8, 11]. As a matter of a fact, multiple in vitro and in silico studies have demonstrated that C-4 molecular area is suitable to lodge significant structural varieties [10, 14]. These efforts were crowned by the preparation and introduction of potent derivatives such as the highly prescribed anticancer drugs etoposide, teniposide, and the prodrug etopophos (Figure 1) [15].

It is worth mentioning that the cytotoxic mechanism of these derivatives is linked to their inhibition of topoisomerase II (topo II), inducing cell death, by enhancing the topo II-mediated DNA cleavage through the stabilization of the transitory DNA/topo II cleavage complex [16]. These agents including etoposide were associated with numerous limitations such as cytotoxicity toward normal cells, drug resistance, and poor bioavailability [17, 18].

**Figure 2:**
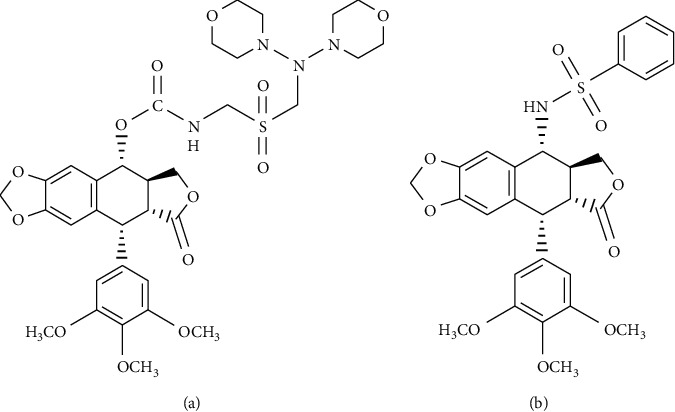
(a) *N*-(aminosulfonyl)-4-podophyllotoxin carbamate derivative; (b) 4-sulphonamide derivatives.

Recently, the design of 4-substituted podophyllotoxin derivatives containing sulfonamide groups was reported [19]. This study exploited the diverse physical, chemical, and biological properties of reported derivatives, in particular, compound (A) in Figure 2; an *N*-(aminosulfonyl)-4-podophyllotoxin carbamate derivative demonstrated high in vitro antiproliferative activity against tumor cell lines such as cervix carcinoma (HeLa), lung carcinoma (A-549), ileocecal carcinoma (HCT-8), and liver carcinoma (HepG2) cell lines, whereas low toxicity against the normal lung (WI-38) cell line. Biological data showed that the derivatives inhibit tubulin polymerization and microtubule assembly in HeLa cells [19]. In another study, 4-sulfonylamino derivatives exemplified by compound B in Figure 2 exerted highly potent cytotoxicity against human prostate (DU145), colon (HT29), breast (MCF7), multidrug-resistant breast (MCF7/ADR), lung (NCI–H460), and brain (U251) cancer cell lines [20]. In addition, a novel series of C-4*β*-disulfide/trisulfide-containing podophyllotoxin derivatives showed good anticancer activity against oral cancer (KB/VCR) cell lines superior to that of etoposide [21]. This work reports the design and synthesis of 4-O-podophyllotoxin sulfamate derivatives having various substituents to afford more potent and less toxic compounds.

## 2. Materials and Methods

### 2.1. Chemicals

All chemicals, reagents, and solvents were of analytical grade and used directly without extra purification. Chloroform, methanol, cyclohexane, ethylacetate, dichloromethane (DCM), and triethylamine (TEA) were purchased from Fisher Scientific (UK) and Tedia Company (USA). Podophyllotoxin, chlorosulfonic acid, ammonia, 2-picolylamine(2-aminomethyl pyridine), 2-(2-aminoethyl)pyridine, 2-amino pyridine, 4-fluoroaniline, and 2-amino anthracene were purchased from Sigma-Aldrich (Germany).

### 2.2. Instruments

Nuclear Magnetic Resonance (NMR) charts were recorded by BRUKER 500 MHz Avance III. Chemical shifts are reported in ppm related to tetramethylsilane (TMS), internal standard. Deuterated chloroform (CDCl_3_) was used as a solvent in sample preparation. ^1^H-NMR data are reported as following: chemical shift (ppm), multiplicity, coupling constant (Hz), number of protons, and their corresponding proton(s). For initial identification of compounds, infrared (IR) spectra were recorded using Shimadzu 8400 FT-IR spectrophotometer (Japan). All tested compounds were triturated with potassium bromide (KBr) and compressed into thin film discs (ACros, Belgium). The melting point (m.p) was measured using Gallenkamp melting point apparatus (Gallenkamp, UK). Thin layer chromatography (TLC) was performed on 20 × 20 cm aluminum plates precoated fluorescent silica gel GF254 (ALBET, Germany). The TLC was visualized under UV lamp, Spectroline® cabinet, Model CX-20 (USA), at 254 and/or 360 nm. For the efficient and gentle removal of solvents from the samples, Rotavapor model R-114 (Buchi, Switzerland) was used.

### 2.3. Synthesis

#### 2.3.1. General Procedure for the Synthesis of Compounds 2–7

**Scheme 1:**
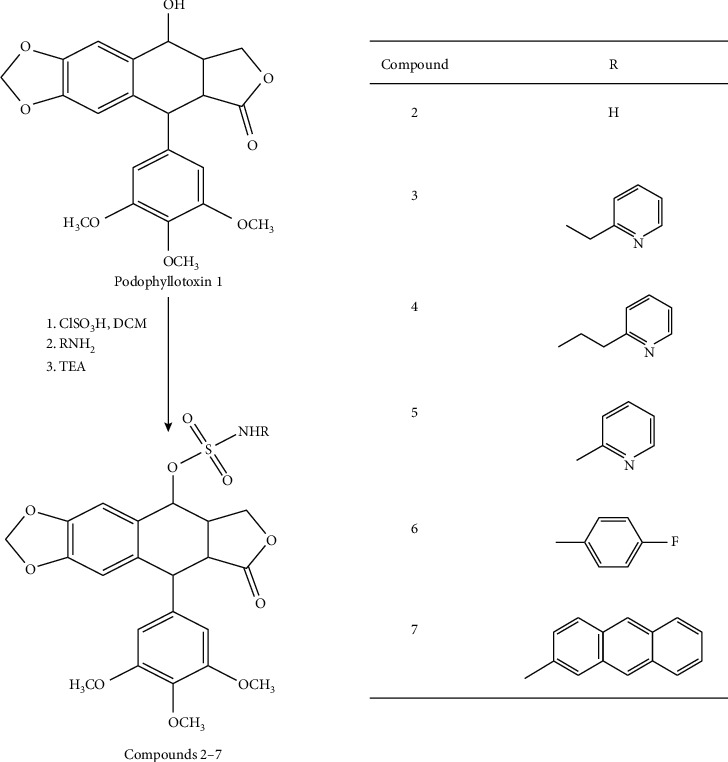
Synthesis of compounds 2–7.

To a stirred solution of **1** (1.0 mmol) in dry dichloromethane (CH_2_Cl_2_) (10 mL) cooled in ice bath, chlorosulfonic acid (ClSO_3_H) (1.5 mmol) solution in dry CH_2_Cl_2_ (10 mL) was added cautiously drop wise. After stirring for 1 h at room temperature, ammonia or aryl amines (2.0 mmol) were added. The mixture was reacted for 1–4 h at room temperature. Then, triethylamine (TEA) (3.0 mmol) was added and filtered. The filtrate was evaporated to dryness, and the crude residue was purified by column chromatography on silica gel with cyclohexane-ethylacetate to afford compounds **2–7,**Scheme 1.

#### 2.3.2. 4-O-Podophyllotoxin Sulfamate (2)

White powder (44%); m.p 234°C; *R*_*f*_ = 0.7 in (chloroform: methanol, 98 : 2); ^1^H-NMR (400 Hz, CDCl_3_): *δ* = 3.23 (m, 1H), 3.74 (s, 6H, 2xOCH_3_), 3.80 (s, 3H, OCH_3_), 4.60 (m, 1H), 5.70 (m.1H), 4.77 (d, *J* = 5.20 Hz, 1H), 5.95 (br *s*, 2H, NH_2_), 6.20 (d, *J* = 7.20 Hz, 2H), 6.35 (s, 2H, methylenedioxy), 6.50 (s, 1H, Ar–H), 7.17 (s, 2H, Ar–H), 7.32 (s, 1H, Ar–H) ppm; ^13^C-NMR (CDCl_3_): *δ* = 29.7 (1C), 45.0 (1C), 46.5 (1C), 50.6 (1C), 56.0 (1C), 56.3 (2C), 60.9 (1C), 62.2 (1C), 101.2 (1C), 106.1 (2C), 106.6 (1C), 112.0 (1C), 128, 8 (1C), 134.5 (1C), 140.0 (1C), 146.1 (1C), 147.6 (1C), 150.1 (1C), 153.3 (1C), 178.4 (1C) ppm; IR (KBr disc): *ν* = 3350 (NH_2_ sulfamate), 1730 (CO-g-lactone), 1600 (OSO_2_), 1430 (OSO_2_) cm^−1^.

#### 2.3.3. 4-*N*-(2-Pyridinylmethyl)podophyllotoxin Sulfamate (3)

White powder (31%); m.p 230°C; *R*_*f*_ = 0.75 in (chloroform: methanol, 98 : 2); ^1^H-NMR (400 Hz, CDCl_3_): *δ* = 3.0 (s, 2H), 3.15 (m, 1H), 3.74 (s, 6H, 2xOCH_3_), 3.80 (s, 3H, OCH_3_), 4.60 (m, 1H), 5.55 (m, 1H), 4.77 (d, *J* = 5.18 Hz, 1H), 5.75 (br *s*, 1H, NH), 6, 15 (m, 1H), 6.20 (d, *J* = 7.15 Hz, 2H), 6.25 (m, 1H), 6.35 (s, 2H, methylenedioxy), 6.50 (s, 1H, Ar–H), 7.07 (s, 2H, Ar–H), 7.15 (s, 1H, Ar–H), 7.40 (m, 1H), 7, 45 (m, 1H) ppm; ^13^C-NMR (CDCl_3_): *δ* = 24 (1C), 29.7 (1C), 45.0 (1C), 46.5 (1C), 50.6 (1C), 56.2 (1C), 57.9 (2C), 60.9 (1C), 67.4 (1C), 101.5 (1C), 108.3 (2C), 109.6 (1C), 111.0 (1C), 128,8 (1C), 134.5 (1C), 137.2 (1C), 140.0 (1C), 143.6 (1C), 146.1 (1C), 147.6 (1C), 148.2 (1C), 150.1 (1C), 152.5 (1C), 153, 1 (1C), 154.0 (1C), 175.0 (1C) ppm; IR (KBr disc): *ν* = 3310 (NH_2_ sulfamate), 1710 (CO-g-lactone), 1570 (OSO_2_), 1410 (OSO_2_) cm^−1^.

#### 2.3.4. 4-*N*-(2-Pyridinylethyl)podophyllotoxin Sulfamate (4)

White powder (20%); m.p 225°C; *R*_*f*_ = 0.8 in (chloroform: methanol, 98 : 2); ^1^H-NMR (400 Hz, CDCl_3_)): *δ* = 2.05 (m, 2H), 2.78 (m, 2H), 3.05 (m, 1H), 3.74 (s, 6H, 2xOCH_3_), 3.80 (s, 3H, OCH_3_), 4.50 (m, 1H), 5.25 (m.1H), 4.77 (m, 1H), 5.75 (br *s*, 1H, NH), 6, 10 (m, 1H), 6.15 (m, 2H), 6.17 (m, 1H), 6.30 (s, 2H, methylenedioxy), 6.41 (s, 1H, Ar–H), 6.90 (s, 2H, Ar–H), 7.0 (s, 1H, Ar–H), 7.23 (m, 1H), 7, 30 (m, 1H) ppm; ^13^C-NMR (CDCl_3_): *δ* = 14.1 (1C), 22.7 (1C), 29.2 (1C), 42.8 (1C), 46.1 (1C), 50.3 (1C), 56.2 (1C), 56.3 (2C), 60.8 (1C), 67.1 (1C), 101.3 (1C), 107.7 (2C), 109.6 (1C), 111.0 (1C), 128.8 (1C), 134.5 (1C), 137.2 (1C), 140.0 (1C), 143.6 (1C), 146.1 (1C), 147.3 (1C), 148.0 (1C), 150.1 (1C), 152.3 (1C), 153.0 (1C), 153.2 (1C), 167.0 (1C) ppm; IR (KBr disc): *ν* = 3300 (NH_2_ sulfamate), 1700 (CO-g-lactone), 1566 (OSO_2_), 1410 (OSO_2_) cm^−1^

#### 2.3.5. 4-*N*-(2-Pyridinyl)podophyllotoxin Sulfamate (5)

White powder (35%); m.p 230°C decomp.; *R*_*f*_ = 0.73 in (chloroform: methanol, 98 : 2); ^1^H-NMR (400 Hz, CDCl_3_): *δ* = 3.32 (m, 1H), 3.80 (s, 6H, 2xOCH_3_), 3.85 (s, 3H, OCH_3_), 4.70 (m, 1H), 5.95 (m.1H), 4.77 (m, 1H), 6.55 (br *s*, 1H, NH), 6.27 (m, 1H), 6.32 (m, 2H), 6.35 (m, 1H), 6.43 (s, 2H, methylenedioxy), 6.65 (s, 1H, Ar–H), 7.20 (s, 2H, Ar–H), 7.30 (s, 1H, Ar–H), 7.64 (m, 1H), 7, 75 (m, 1H) ppm; ^13^C-NMR (CDCl_3_): *δ* = 31 (1C), 45.5 (1C), 46.9 (1C), 52 (1C), 57 (1C), 58.7 (2C), 63 (1C), 68.2 (1C), 101.7 (1C), 108.8 (2C), 110.7 (1C), 112.0 (1C), 129.4 (1C), 134.9 (1C), 137.7 (1C), 140.6 (1C), 144.4 (1C), 147 (1C), 148.3 (1C), 149 (1C), 150.4 (1C), 153.3 (1C), 154 (1C), 154.7 (1C), 177.2 (1C) ppm; IR (KBr disc): *ν* = 3390 (NH_2_ sulfamate), 1730 (CO-g-lactone), 1620 (OSO_2_), 1460 (OSO_2_) cm^−1^.

#### 2.3.6. 4-*N*-(4-Flourophenyl)podophyllotoxin Sulfamate (6)

White powder (20%); m.p 220°C; *R*_*f*_ = 0.8 in (chloroform: methanol, 98 : 2); ^1^H-NMR (400 Hz, CDCl_3_): *δ* = 3.0 (m, 1H), 3.76 (s, 6H, 2xOCH_3_), 3.82 (s, 3H, OCH_3_), 4.64 (m, 1H), 5.62 (m.1H), 4.74 (m, 1H), 5.95 (br *s*, 1H, NH), 6.30 (m, 1H), 6.27 (d, *J* = 5.30 Hz, 2H), 6.32 (d, *J* = 6.10 Hz, 2H, Ar–H), 6.40 (s, 2H, methylenedioxy), 6.55 (d, *J* = 6.15 Hz, 2H, Ar–H), 7.10 (s, 2H, Ar–H), 7.21 (s, 1H, Ar–H) ppm; ^13^C-NMR (CDCl_3_): *δ* = 30.7 (1C), 45.4 (1C), 46.8 (1C), 51 (1C), 56.6 (1C), 58.3 (2C), 61.2 (1C), 67.8 (1C), 102.3 (1C), 108.6 (2C), 109.9 (1C), 111.3 (1C), 129.2 (1C), 134.8 (1C), 137.6 (1C), 140.9 (1C), 144 (1C), 146.5 (1C), 148.2 (1C), 148.7 (1C), 150.1 (1C), 152.7 (1C), 153.4 (1C), 154.3 (1C), 156.7 (1C), 176.0 (1C) ppm; IR (KBr disc): *ν* = 3330 (NH_2_ sulfamate), 1710 (CO-g-lactone), 1570 (OSO_2_), 1430 (OSO_2_) cm^−1^. This compound was unstable, and it was excluded from the biological assay.

#### 2.3.7. 4-*N*-(2-Anthracenyl)podophyllotoxin Sulfamate (7)

White powder (58%); m.p 246°C decomp.; *R*_*f*_ = 0.89 in (chloroform: methanol, 98 : 2); ^1^H-NMR (400 Hz CDCl_3_)): *δ* = 3.08 (m, 1H), 3.77 (s, 6H, 2xOCH_3_), 3.81 (s, 3H, OCH_3_), 4.64 (m, 1H), 4.89 (m.1H), 3.81 (m, 1H), 5.96 (br *s*, 1H, NH), 4.64 (m, 2H), 6.25 (m, 1H, Ar–H), 6.35 (s, 2H, methylenedioxy), 6.55 (s, 1H), 6.83 (m, 2H, Ar–H), 7.09 (s, 1H), 7.43 (m, 1H), 7.52 (m, 2H), 7.82 (m, 1H), 7.91 (m, 2H), 8.11 (s, 1H, Ar–H), 8.26 (s, 1H, Ar–H) ppm; ^13^C-NMR (CDCl_3_): *δ* = 29.7 (1C), 45.9 (1C), 46.2 (1C), 50.3 (1C), 56.3 (1C), 58.0 (2C), 60.8 (1C), 69.0 (1C), 101.6 (1C), 108.4 (2C), 109.6 (1C), 110.8 (1C), 105.6 (1C), 120.9 (1C), 121 (1C), 123 (1C), 125.1 (1C), 125.8 (1C), 126, 5 (1C), 127 (1C), 128.4 (1C), 128.9 (1C), 129.2 (1C), 131.9 (1C), 133.6 (1C), 134.8 (1C), 137.6 (1C), 146.5 (1C), 148.2 (1C), 148.7 (1C), 150.1 (1C), 152.4 (1C), 152.9 (1C), 171.0 (1C) ppm; IR (KBr disc): *ν* = 3310 (NH_2_ sulfamate), 1680 (CO-g-lactone), 1575 (OSO_2_), 1410 (OSO_2_) cm^−1^.

### 2.4. Cell Culture

Three cancer cell lines, human breast adenocarcinoma (MCF7), human ovary adenocarcinoma (A2780), and human colon adenocarcinoma (HT29), in addition the normal human fetal lung fibroblast (MRC5), were used. All cell lines were obtained from the ATCC. The three cancer cells were subcultured in RPMI-1640 media (10% FBS), while MRC5 was maintained in Eagles minimum essential medium (EMEM, 10% FBS), all at 37°C, 5% CO_2_, and 100% relative humidity [22].

### 2.5. Cytotoxicity Assay

As previously reported [23, 24], the cytotoxicity of the six compounds, including podophyllotoxin as reference, was evaluated by the MTT assay. The three cell lines and one normal fibroblast cells were separately cultured in 96-well (3 × 10^3^/well) and incubated at 37°C overnight. Final compound concentrations are as follows: 0, 0.005, 0.050, 0.500, 5.000, and 25.000 *μ*M (DMSO 0.1%; *n* = 3). Plates were incubated for 72 h, followed by addition of MTT to each well. Plates were incubated for 3 hr, supernatant was aspirated, and DMSO was added to each well. The absorbance was read on multiplate reader. The optical density of the purple formazan A_550_ is proportional to the number of viable cells. Compound concentration causing 50% inhibition (IC_50_) compared to control cell growth (100%) was determined. GraphPad Prism version 5.00 for Windows, GraphPad Software, San Diego California, USA, was used for analysis.

### 2.6. Annexin V FITC/PI Apoptosis Assay

Annexin V FITC/PI assay was used to evaluate possible ability to induce apoptosis [25]. MCF7 cells were cultured in 6 well plates (1 × 10^5^ cells/well) overnight at 37°C. Compounds **2**, **3**, and **7** were used to treat cells (0, 0.5, 1.0, and 5.0 *µ*M). After 24 h, the supernatant of treated cells was collected in tubes and kept in ice, and cells were trypsinized and incubated at 37°C before being added to the tubes. MCF7 cells were centrifuged (2000 rpm) and washed with PBS (x1), and pellets resuspended in the binding buffer (100 *μ*L) and annexin V FITC (10 *μ*L). Tubes were incubated at room temperature in dark for 20 min, before adding binding buffer (400 *μ*L) and 10 *μ*L propidium iodide (PI). Analysis was performed by flow cytometry (BC, FC500, USA). Different cell populations (early apoptotic, late apoptotic, and necrotic cells) were identified by annexin V and PI staining.

## 3. Results and Discussion

### 3.1. Chemistry

The synthesis of podophyllotoxin sulfamates, compounds **2–7,** was achieved by reaction of podophyllotoxin (**1**) with chlorosulfonic acid in dry dichloromethane (DCM) (Scheme 1). Then, the podophyllotoxin sulfonyl chloride intermediate derivatives were immediately treated with ammonia or with the corresponding aryl and heteroaryl amines in the presence of triethylamine (TEA). The structures of the target compounds **2–7** were identified by IR, ^1^H-NMR, and ^13^C-NMR spectral analysis.

### 3.2. Cytotoxicity Assay

The cytotoxicity of the new podophyllotoxin derivatives against breast, ovarian, and colon cancer cells was evaluated using MTT assay. Additionally, one normal cell line was tested to assess the selectivity of the new derivatives. Compound **3** shows the highest activity against the three cell lines (0.150–0.220 *µ*M), and it was 9.1–13.5-fold more selective against MCF7, A2780, and HT29 compared to MRC5 cells. Compound **2** also showed ≤1 *µ*M activity against MCF7, A2780, and HT29, and it was less cytotoxic against MRC5 cells. Compounds **4** and **5** showed similar activity to **3**, but they showed no selectivity against MRC5. Finally, compound **7** was the least active (IC_50_ : 2.120–5.082 *µ*M), but it showed 2–4-fold selectivity against the normal cells. Compound **3** showed the comparable activity to podophyllotoxin (Table 1).

### 3.3. Annexin V FITC/PI Apoptosis Assay

**Figure 3:**
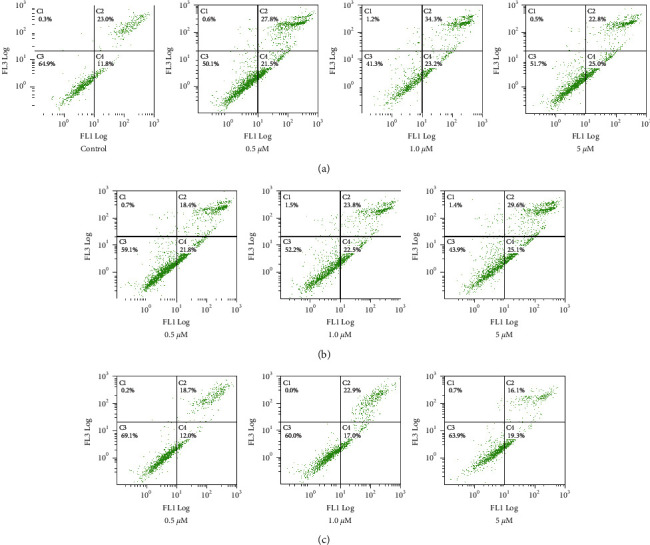
Histogram showing different phases of staining MCF7 cells with annexin V FITC/PI treated with vehicle control. Compounds (a) 2, (b) 3, and (c) 7, each in three concentrations, 24 h. *X*-axis: annexin (V); *y*-axis: PI. C1: (necrosis death, PI+/annexin V-); C2: (late apoptosis, PI+/annexin V+); C3: (living cells, PI-/annexin V-); C4: (early apoptosis, PI-/annexin V+). Experiment was repeated 2x.

Compounds **2**, **3**, and 7 were selected for further investigations to explain their mechanism of action. Annexin V FITC/PI assay was used to evaluate whether they can induce apoptosis or not in MCF7 cells, following 24 h treatment. Compounds **2**, **3**, and **7** increased the early apoptotic MCF7 cell populations in a dose-dependent manner (1.7–2.2-fold, compared to control), as shown in Figure 3.

The desired podophyllotoxin sulfamate derivatives were successfully prepared in a one-pot reaction in good yields. This novel method was developed in our lab applying the coupling reaction between the alcohol podophyllotoxin with excess chlorosulfonic acid affording a highly reactive O-sulfonyl chloride intermediates that are immediately subjected to sulfonamidation reactions with ammonia or amino/heteroaryl derivatives. The obtained compounds were identified by IR and ^1^H and ^13^C-NMR spectral data. The prepared compounds were evaluated for their cytotoxic activity against MCF7, A2780, and HT29, in addition to MRC5. All tested compounds showed cytotoxicity in the micromolar range but were less potent than podophyllotoxin, which was used as a reference. Compound **3** was the most potent derivative, and it exhibited a good selectivity margin; in fact, it showed 9.1–13.5 times more cytotoxicity in cancer cells compared to normal MRC5 cells. Compounds **2** and 7 showed cytotoxicity with moderate selectivity. Consequently, compounds 2, 3, and 7 were also evaluated for their apoptotic inducing ability on MCF7 cells and showed activity in a dose-dependent manner that partially explains the mechanism of their activity. These results indicate that podophyllotoxin sulfamate derivatives are valid candidates for further development as anticancer agents.

## 4. Conclusion

Derivatives of 4-O-podophyllotoxin sulfamate were synthesized through a simple and direct one-pot methodology developed in our laboratory. The synthesized derivatives showed cytotoxicity in the micromolar concentrations. Compounds (**2**, **3**, and **7**) showed a dose dependent increase in apoptotic events. Yet, the activity of all compounds was inferior to that of podophyllotoxin. We are looking for optimizing the core structure of this scaffold and exploring the activity of these derivatives *in vivo* study to get insights about the efficacy and tolerability in animal models.

## Figures and Tables

**Table 1:** Cytotoxic activity of the verified compounds (2, 3, 4, 5, and 7) (MTT 72 h, IC50 ± SD *μ*M).

Compound	MCF7	A2780	HT29	MRC5
2	0.648 ± 0.087	0.729 ± 0.363	1.343 ± 0.637	7.507 ± 1.510
3	0.150 ± 0.060^*∗*^	0.179 ± 0.010^*∗*^	0.222 ± 0.098^*∗*^	2.027 ± 0.250
4	0.212 ± 0.010^*∗*^	0.456 ± 0.180^*∗*^	0.228 ± 0.075^*∗*^	0.048 ± 0.003^*∗∗*^
5	0.184 ± 0.052^*∗*^	0.132 ± 0.012^*∗*^	1.048 ± 0.005	0.047 ± 0.042^*∗∗*^
7	2.120 ± 0.989	3.652 ± 1.939	5.082 ± 0.673	9.076 ± 1.010
Podophyllotoxin	0.004 ± 0.001^*∗∗∗*^	0.007 ± 0.001^*∗∗∗*^	0.002 ± 0.001^*∗∗∗*^	0.043 ± 0.060^*∗∗*^

Data are representation of three independent experiments (*n* = 4). Statistical difference (one-way ANOVA, Tukey's post hoc): *p* < 0.05^*∗*^, *p* < 0.01^*∗∗*^, and *p* < 0.001^*∗∗∗*^ were considered significant.

## Data Availability

Data used to support the findings of this study are available from the corresponding author upon request.

## Supplementary Materials

Supplementary Materials
^1^H-NMR spectrum (500 MHz, CDCl_3_) of podophyllotoxin and its derivatives: 2–7. Page 2: ^1^H-NMR of compound 1: podophyllotoxin. Page 3: ^1^H-NMR of compound 2: 4-O-podophyllotoxin sulfamate. Page 4: ^1^H-NMR of compound 3: 4-*N*-(2-pyridinylmethyl)-podophyllotoxin sulfamate. Page 5: ^1^H-NMR of compound 4: 4-*N-*(2-pyridinylethyl)-podophyllotoxin sulfamate. Page 6: ^1^H-NMR of compound 5: 4-*N-*(2-pyridinyl)-podophyllotoxin sulfamate. Page 7: ^1^H-NMR of compound 6: 4-*N-*(4-flourophenyl)-podophyllotoxin sulfamate. Page 8: ^1^H-NMR of compound 7: 4-*N*-(2-anthracenyl)-podophyllotoxin sulfamate.
