# Supplementary material for: Design and Synthesis of 4-O-Podophyllotoxin Sulfamate Derivatives as Potential Cytotoxic Agents
Source: Evid Based Complement Alternat Med. 2021 Jan 25;2021:6672807. doi: 10.1155/2021/6672807 (PMC7857870; doi:10.1155/2021/6672807)
Supplement: Supplementary Materials — 1H-NMR spectrum (500 MHz, CDCl3) of podophyllotoxin and its derivatives: 2–7. Page 2: 1H-NMR of compound 1: podophyllotoxin. Page 3: 1H-NMR of compound 2: 4-O-podophyllotoxin sulfamate. Page 4: 1H-NMR of compound 3: 4-N-(2-pyridinylmethyl)-podophyllotoxin sulfamate. Page 5: 1H-NMR of compound 4: 4-N-(2-pyridinylethyl)-podophyllotoxin sulfamate. Page 6: 1H-NMR of compound 5: 4-N-(2-pyridinyl)-podophyllotoxin sulfamate. Page 7: 1H-NMR of compound 6: 4-N-(4-flourophenyl)-podophyllotoxin sulfamate. Page 8: 1H-NMR of compound 7: 4-N-(2-anthracenyl)-podophyllotoxin sulfamate. [file 6672807.f1.pdf]

Design and Synthesis of 4-*O*-Podophyllotoxin Sulfamate Derivatives as Potential Cytotoxic Agents.

Ammar Bader[1], Majdi M. Bkhaitan[1, 2], Ashraf N. Abdalla[1], Qasem M. A. Abdallah[3,4], Hamed I. Ali[5], Dima A. Sabbah[6], Ghadeer Albadawi[6], Ghassan M. Abushaikha[7].

[1] Faculty of Pharmacy, Umm Al-Qura University, 21955 Makkah, Saudi Arabia
[2] Biomedical Sciences Unit, Faculty of Medicine, Arab American University, Jenin, Palestine
[3]Department of Pharmacology and Toxicology, College of Pharmacy, Taif University, Taif, Makkah, 21974, Saudi Arabia
[4]Department of Pharmacology & Biomedical Sciences, Faculty of Pharmacy and Medical Sciences, University of Petra, Amman, Jordan
[5]Rangel College of Pharmacy, Health Science Center, Texas A&M University, Kingsville, TX 78363, United States.
[6]Department of Pharmacy, Faculty of Pharmacy, Al-Zaytoonah University of Jordan, P.O. Box 130 Amman 11733 Jordan
[7]Visiting Professor Scholar, Skaggs School of Pharmacy and Pharmaceutical Science, University of California, San Diego.9500 Gilman Drive MC0756, La Jolla, CA 92093-0756, USA

Supplementary section

Page 2: ^{1}H-NMR of compound 1: Podophyllotoxin.
Page 3: ^{1}H-NMR of compound 2: 4-*O*-Podophyllotoxin sulfamate.
Page 4: ^{1}H-NMR of compound 3: 4-*N*-(2-Pyridinylmethyl)-podophyllotoxin sulfamate.
Page 5: ^{1}H-NMR of compound 4: 4-*N*-(2-Pyridinylethyl)-podophyllotoxin sulfamate.
Page 6: ^{1}H-NMR of compound 5: 4-*N*-(2-Pyridinyl)-podophyllotoxin sulfamate.
Page 7: ^{1}H-NMR of compound 6: 4-*N*-(4-Flourophenyl)-podophyllotoxin sulfamate.
Page 8: ^{1}H-NMR of compound 7: 4-*N*-(2-Anthracenyl)-podophyllotoxin sulfamate.

compound 1 (podophyllotoxin)

PROTON NMR

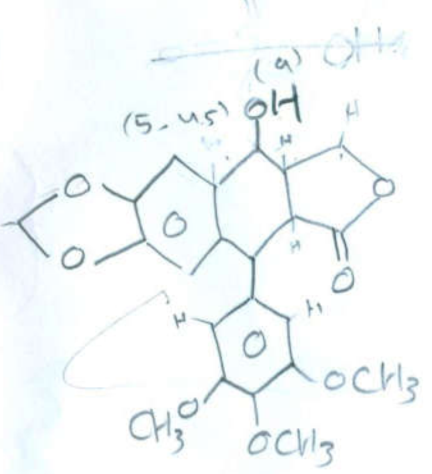

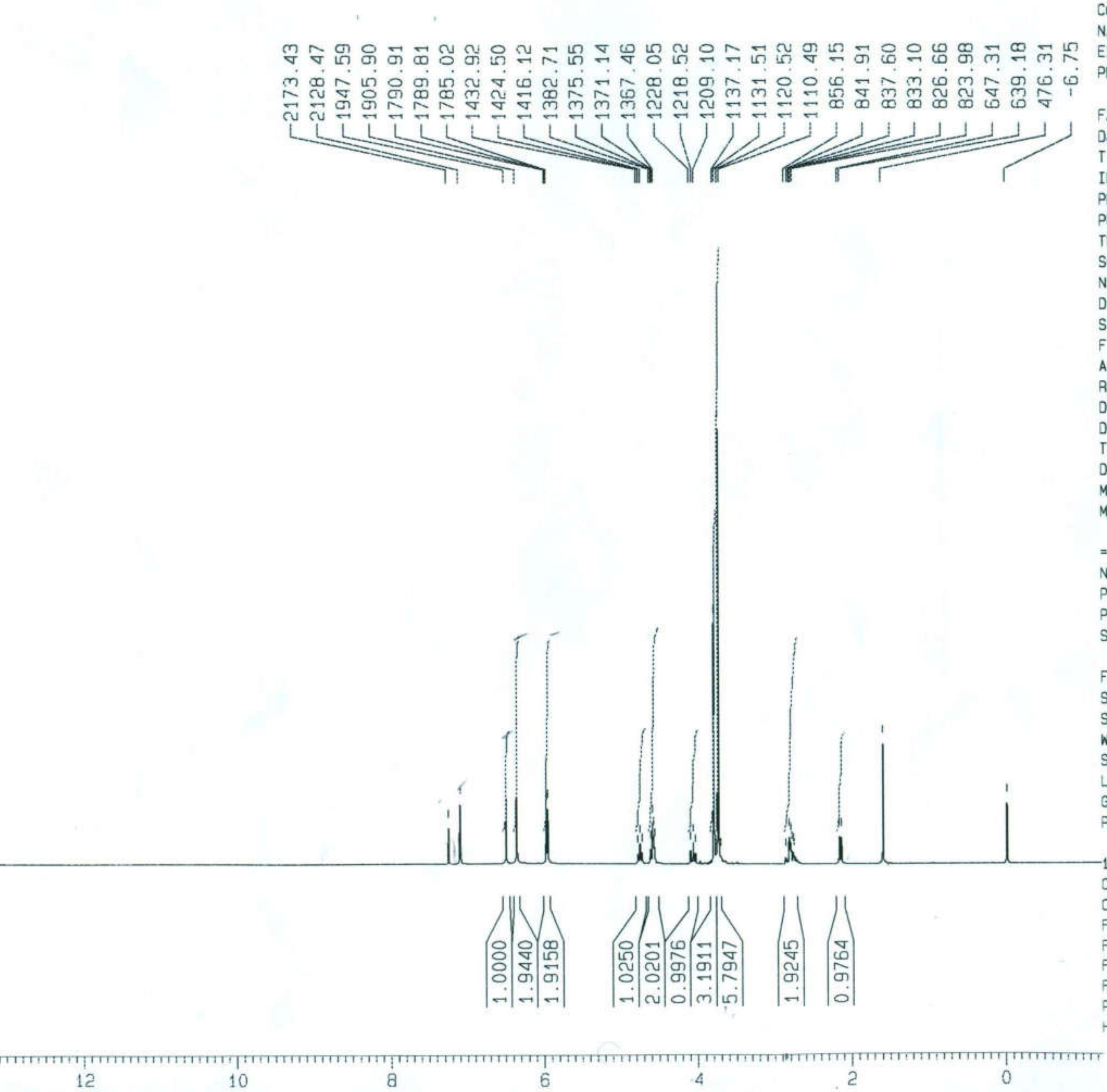

Current Data Parameters
NAME 15nov01aba
EXPNO 591
PROCNO 1

F2 - Acquisition Parameters
Date_ 20151115
Time 12.53
INSTRUM spect
PROBHD 5 mm Dual 13C/
PULPROG zg
TD 65536
SOLVENT CDCl3
NS 1
DS 0
SWH 5787.037 Hz
FIDRES 0.088303 Hz
AQ 5.6623602 se
RG 1290.2
DW 86.400 us
DE 5.50 us
TE 298.2 K
D1 1.00000000 se
MCREST 0.00000000 se
MCWRK 0.01500000 se

======== CHANNEL f1 ======
NUC1 1H
P1 8.60 us
PL1 -3.20 dB
SFO1 300.1325269 MH

F2 - Processing parameters
SI 32768
SF 300.1300122 MH
WDW EM
SSB 0
LB 0.40 Hz
GB 0
PC 1.00

1D NMR plot parameters
CX 23.00 cm
CY 0.00 cm
F1P 18.020 pp
F1 5408.21 Hz
F2P -1.262 pp
F2 -378.83 Hz
PPMCM 0.83834 pp
HZCM 251.61029 Hz

Compound 2; (4-*O*-Podophyllotoxin sulfamate)

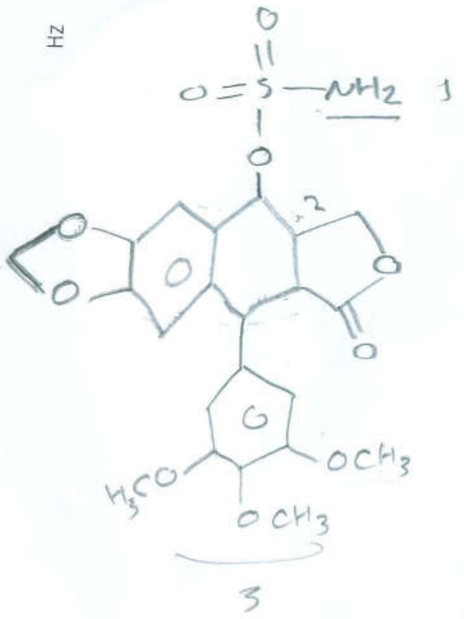

OK

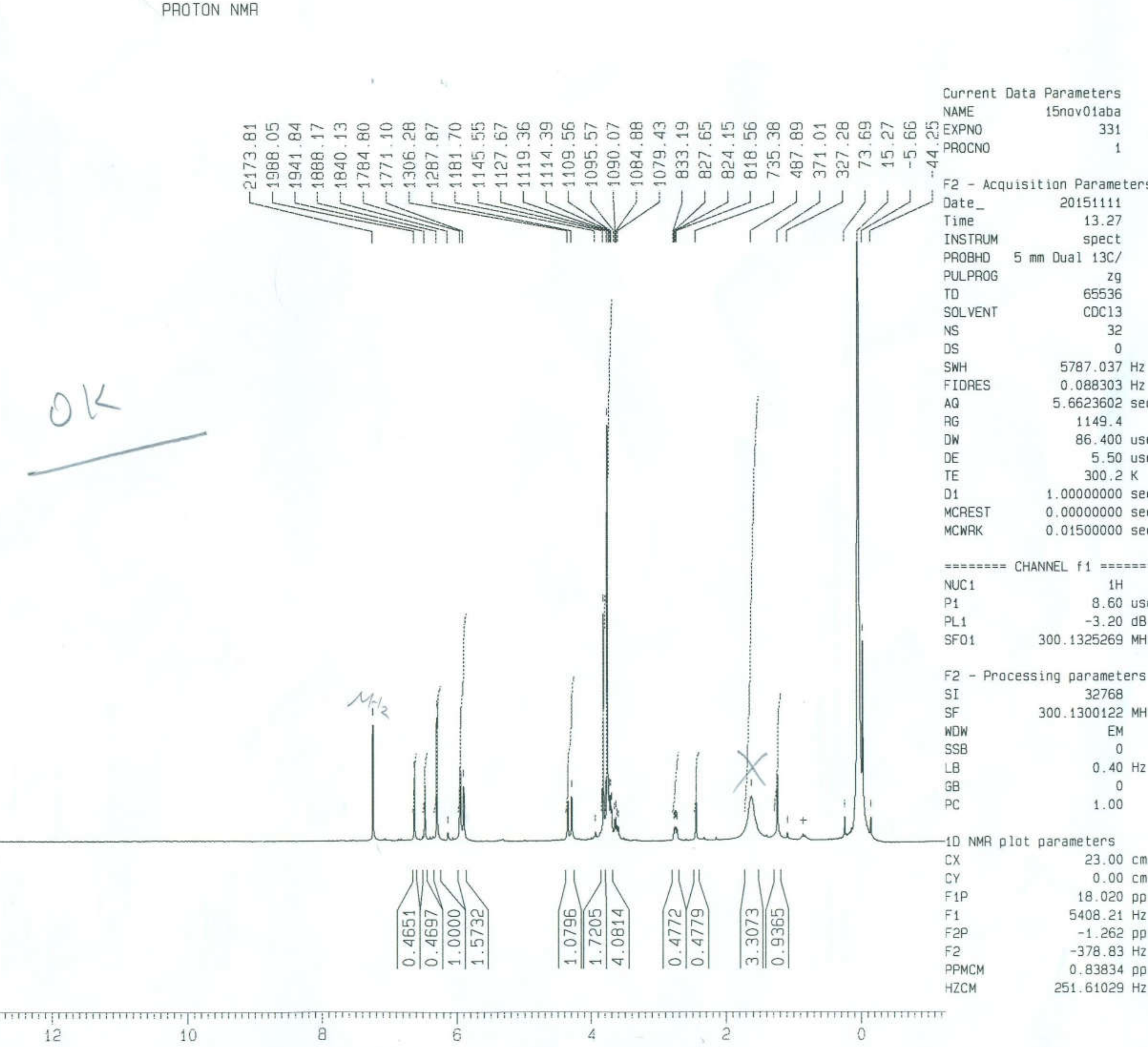

Current Data Parameters	
NAME	15nov01aba
EXPNO	331
PROCNO	1
F2 - Acquisition Parameters	
Date_	20151111
Time	13.27
INSTRUM	spect
PROBHD	5 mm Dual 13C/
PULPROG	zg
TD	65536
SOLVENT	CDCl3
NS	32
DS	0
SWH	5787.037 Hz
FIDRES	0.088303 Hz
AQ	5.6623602 sec
RG	1149.4
DW	86.400 usec
DE	5.50 usec
TE	300.2 K
D1	1.00000000 sec
MCREST	0.00000000 sec
MCWRK	0.01500000 sec
======== CHANNEL f1 ======	
NUC1	1H
P1	8.60 usec
PL1	-3.20 dB
SFO1	300.1325269 MH
F2 - Processing parameters	
SI	32768
SF	300.1300122 MH
WDW	EM
SSB	0
LB	0.40 Hz
GB	0
PC	1.00
1D NMR plot parameters	
CX	23.00 cm
CY	0.00 cm
F1P	18.020 pp
F1	5408.21 Hz
F2P	-1.262 pp
F2	-378.83 Hz
PPMCM	0.83834 pp
HZCM	251.61029 Hz

Compound 3: (4-*N*-(2-Pyridinylmethyl)-podophyllotoxin sulfamate)

PROTON NMR

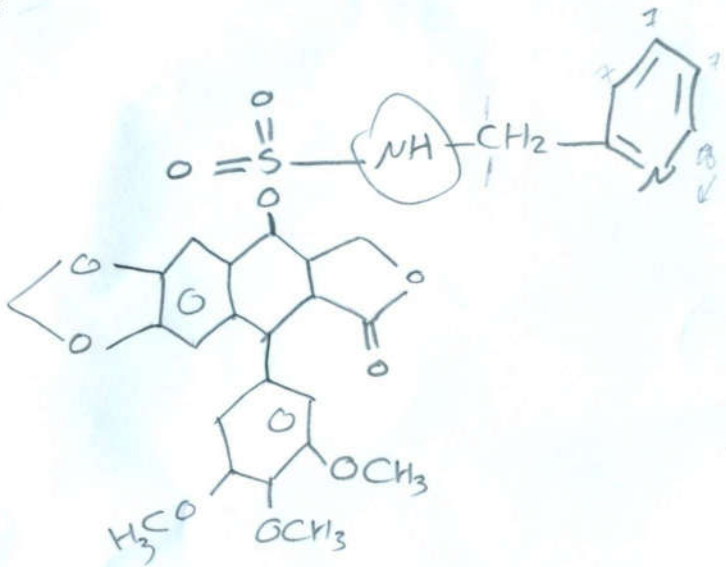

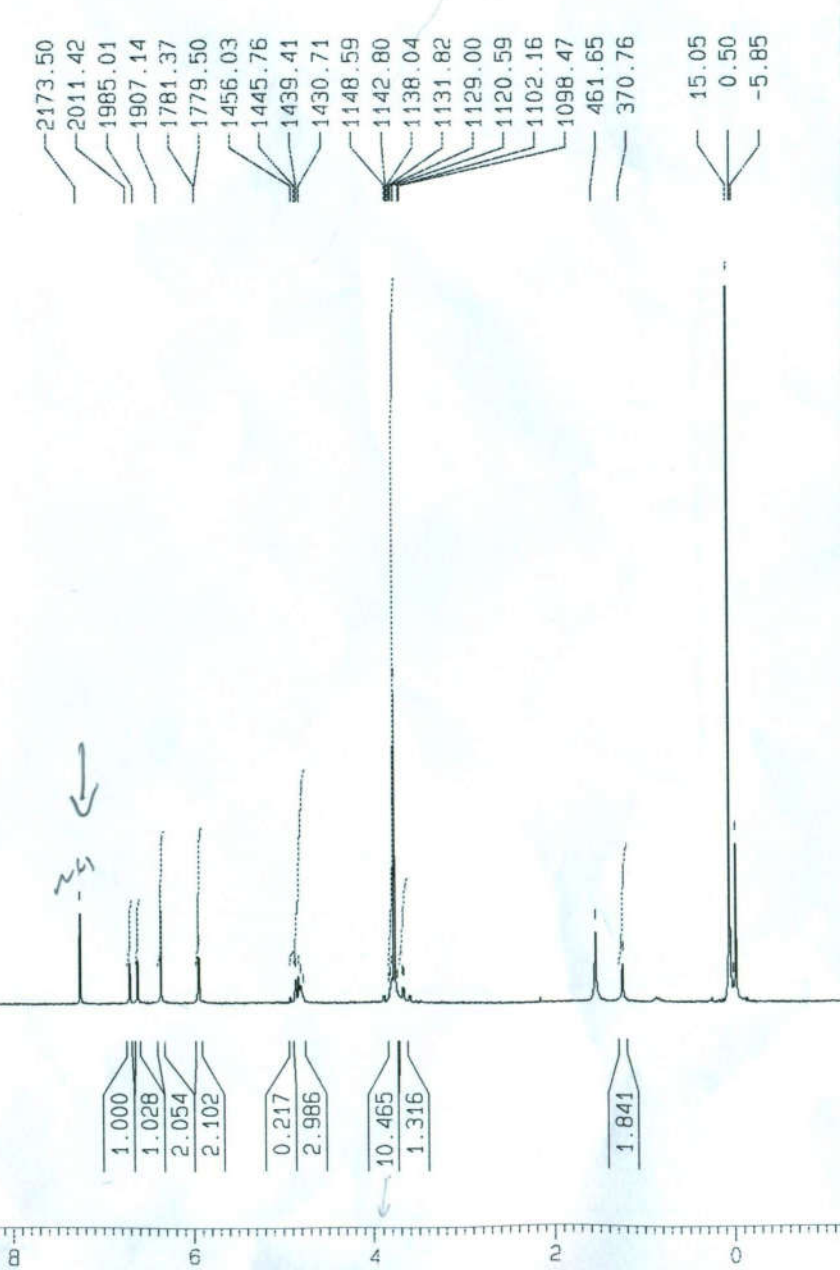

Current Data Parameters
NAME 15nov01aba
EXPNO 581
PROCNO 1

F2 - Acquisition Parameter
Date_ 20151115
Time 12.50
INSTRUM spect
PROBHD 5 mm Dual 13C/
PULPROG zg
TD 65536
SOLVENT CDCl3
NS 1
DS 0
SWH 5787.037 Hz
FIDRES 0.088303 Hz
AQ 5.6623602 se
RG 2298.8
DW 86.400 us
DE 5.50 us
TE 298.2 K
D1 1.00000000 se
MCREST 0.00000000 se
MCWRK 0.01500000 se

======== CHANNEL f1 ======
NUC1 1H
P1 8.60 us
PL1 -3.20 dE
SFO1 300.1325269 MH

F2 - Processing parameters
SI 32768
SF 300.1300122 MH
WDW EM
SSB 0
LB 0.40 Hz
GB 0
PC 1.00

1D NMR plot parameters
CX 23.00 cr
CY 0.00 cr
F1P 18.020 pp
F1 5408.21 Hz
F2P -1.262 pp
F2 -378.83 Hz
PPMCM 0.83834 pp
HZCM 251.61029 H

Compound 4: (4-*N*-(2-Pyridinylethyl)-podophyllotoxin sulfamate)

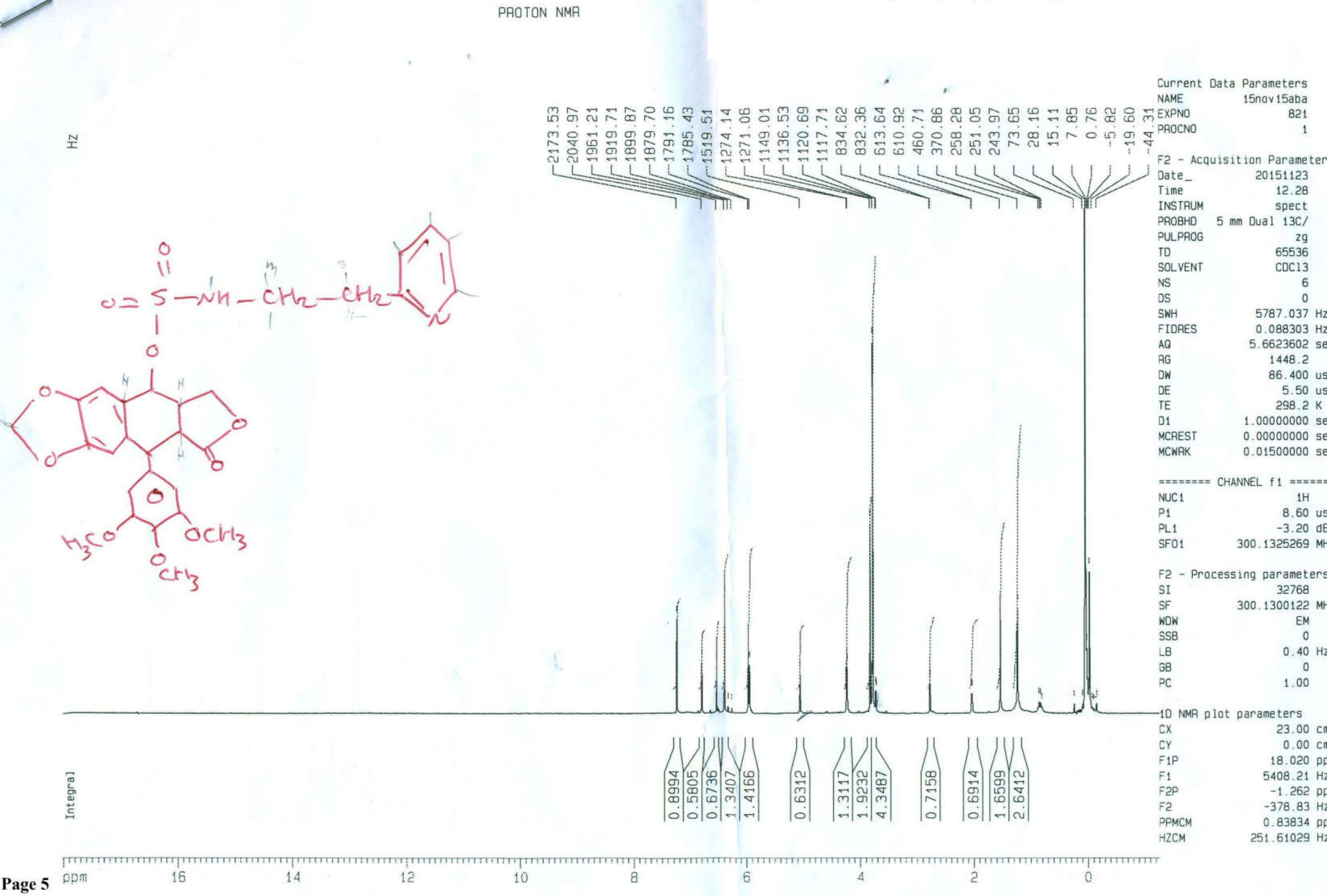

Compound 5: (4-*N*-(2-Pyridinyl)-podophyllotoxin sulfamate)

L4-S2
HNMR

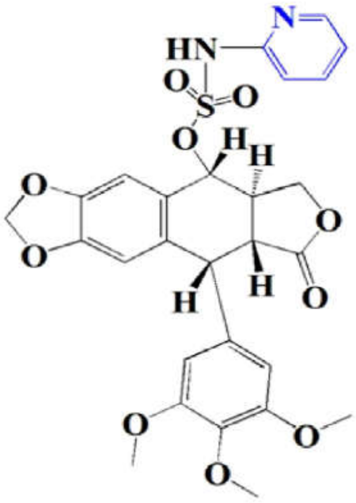

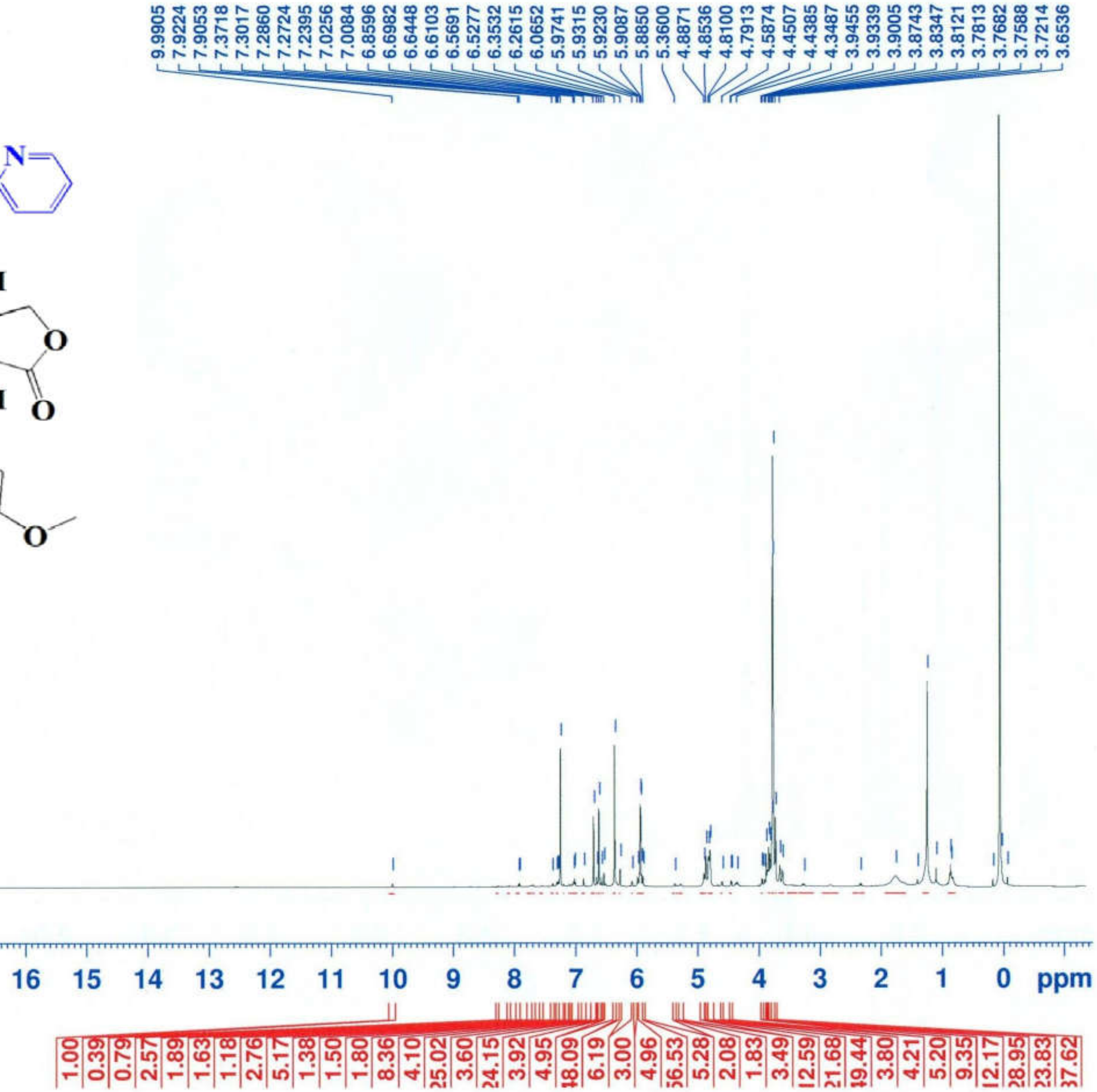

The University of Jordan
Faculty of Science
Department of Chemistry

Instrument Model:
Bruker 500 MHz–Avance III

Operator: Rola Hassouneh
nmr500@ju.edu.jo


```
Current Data Parameters
NAME          15dec15jalal
EXPNO                  541
PROCNO                   1

F2 - Acquisition Parameters
Date_             20160103
Time                  8.47
INSTRUM              spect
PROBHD   5 mm PABBO BB/
PULPROG                 zg
TD                   65536
SOLVENT              CDCl3
NS                      32
DS                       0
SWH            10135.135 Hz
FIDRES          0.154650 Hz
AQ             3.2331092 sec
RG                   49.66
DW                  49.333 usec
DE                    6.50 usec
TE                   300.4 K
D1            2.00000000 sec
TD0                      1

======== CHANNEL f1 ========
SFO1         500.1344108 MHz
NUC1                    1H
P1                   10.60 usec
PLW1         17.39999962 W

F2 - Processing parameters
SI                  131072
SF           500.1300235 MHz
WDW                     EM
SSB      0
LB                    1.00 Hz
GB       0
PC                    1.50
```

Compound 6: (4-*N*-(4-flourophenyl)-podophyllotoxin sulfamate)

L5-S2
HNMR

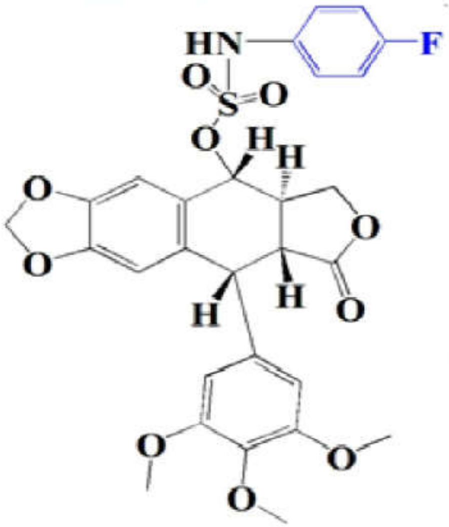

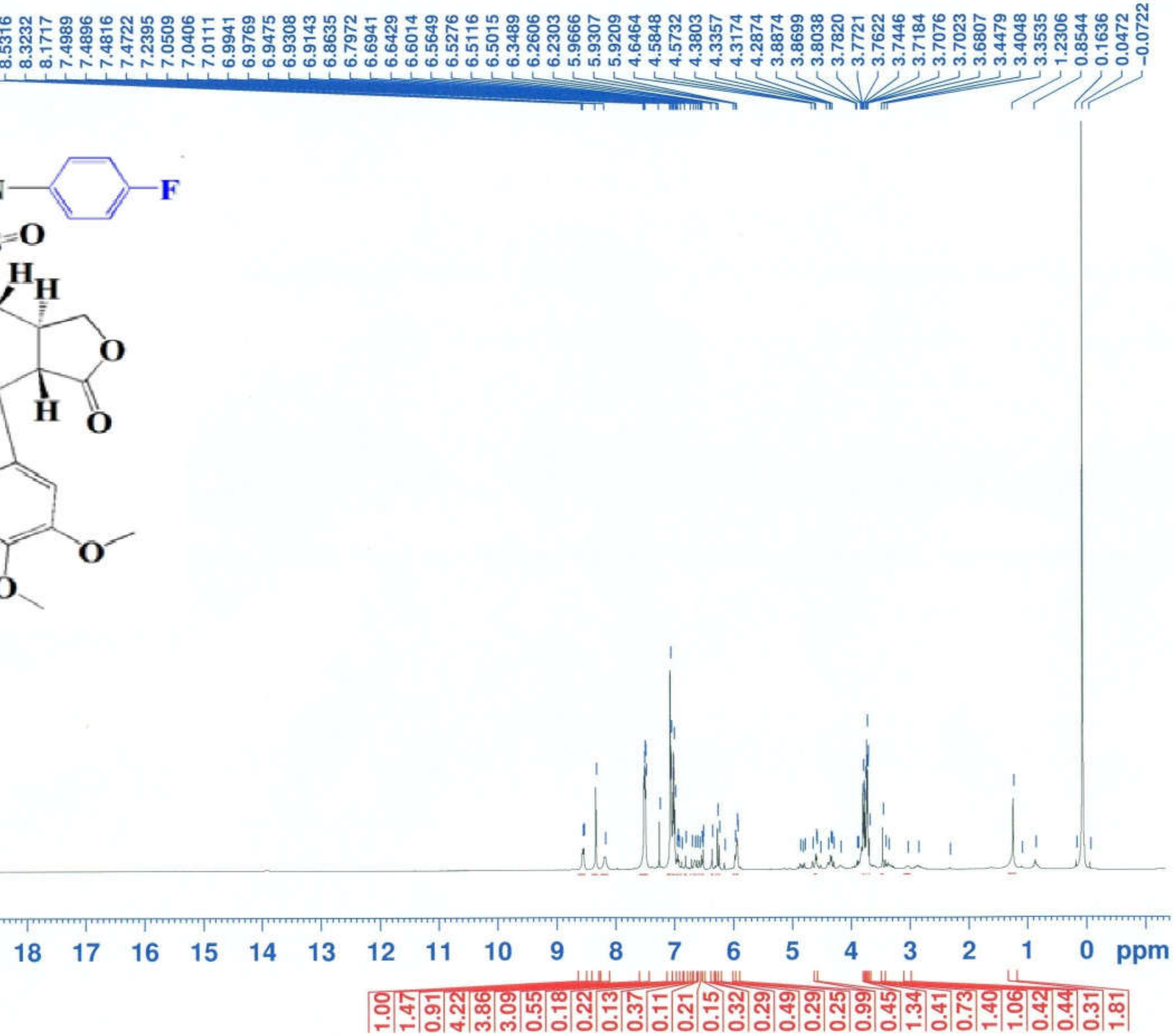

The University of Jordan
Faculty of Science
Department of Chemistry

Instrument Model:
Bruker 500 MHz–Avance III

Operator: Rola Hassouneh
nmr500@ju.edu.jo


```
Current Data Parameters
NAME          15dec15jalal
EXPNO                  511
PROCNO                   1

F2 - Acquisition Parameters
Date_             20151231
Time                  9.46
INSTRUM              spect
PROBHD     5 mm PABBO BB/
PULPROG                 zg
TD                   65536
SOLVENT              CDCl3
NS                      32
DS                       0
SWH              10135.135 Hz
FIDRES            0.154650 Hz
AQ               3.2331092 sec
RG                   35.17
DW                  49.333 usec
DE                    6.50 usec
TE                   300.4 K
D1              2.00000000 sec
TD0                      1

======== CHANNEL f1 ========
SFO1           500.1344108 MHz
NUC1                    1H
P1                   10.60 usec
PLW1           17.39999962 W

F2 - Processing parameters
SI                  131072
SF             500.1300235 MHz
WDW                     EM
SSB      0
LB                    1.00 Hz
GB       0
PC                    1.50
```

Compound 7: (4-*N*-(2-Anthracenyl)-podophyllotoxinsulfamate)

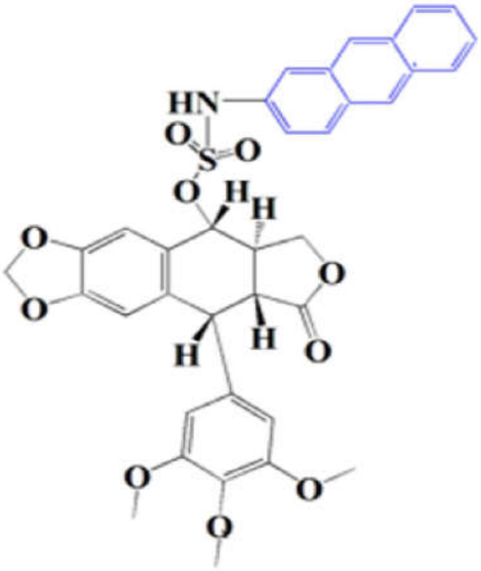

L6
HNMR

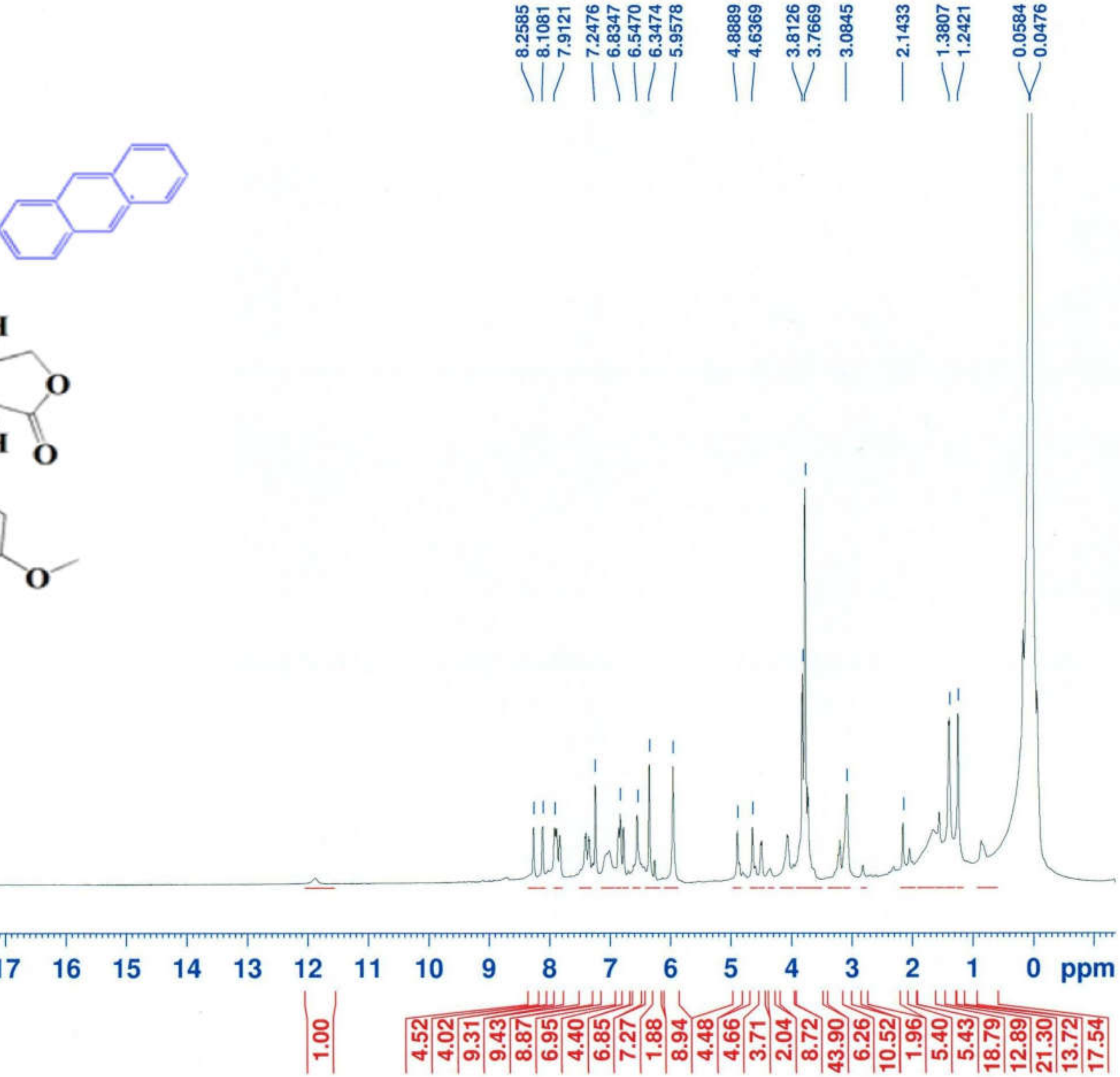

The University of Jordan
Faculty of Science
Department of Chemistry

Instrument Model:
Bruker 500 MHz–Avance III

Operator: Rola Hassouneh
nmr500@ju.edu.jo


```
Current Data Parameters
NAME          16jan15jalal
EXPNO                  131
PROCNO                   1

F2 - Acquisition Parameters
Date_             20160118
Time                 12.46
INSTRUM              spect
PROBHD     5 mm PABBO BB/
PULPROG                 zg
TD                   65536
SOLVENT              CDCl3
NS                      16
DS                       0
SWH              10135.135 Hz
FIDRES            0.154650 Hz
AQ               3.2331092 sec
RG                   71.89
DW                  49.333 usec
DE                    6.50 usec
TE                   300.4 K
D1              2.00000000 sec
TD0                      1

======== CHANNEL f1 ========
SFO1          500.1344108 MHz
NUC1                   1H
P1                   10.60 usec
PLW1           17.39999962 W

F2 - Processing parameters
SI                  131072
SF             500.1300235 MHz
WDW                     EM
SSB      0
LB                    1.00 Hz
GB       0
PC                    2.00
```
